# Research Progress on Avian Influenza Virus and Autophagy: A Review

**DOI:** 10.3390/pathogens15060623

**Published:** 2026-06-11

**Authors:** Zhiqiang Hu, Jiali Li, Ase Hailai, Ran Guan, Xinhong Li, Xi Chen, Yiqun Chen, Mingyu Fan, Zengwen Huang, Guangwen Yan, Chaoyun Yang

**Affiliations:** 1 College of Animal Science, Xichang University, Xichang 615013, China; 2 Key Laboratory of Animal Epidemic Disease Detection and Prevention in Panxi District, Xichang University, Xichang 615013, China; 3 Liangshan Ecological Breeding Engineering Technology Research Center, Xichang University, Xichang 615013, China; 4 College of Veterinary Medicine, Northwest A&F University, Yangling 712100, China

**Keywords:** avian influenza virus, genotypes, autophagy, autophagic flux, host immunity, autophagy-mediated antiviral strategy

## Abstract

Avian influenza virus (AIV), a zoonotic pathogen capable of cross-species transmission, poses a significant global health threat due to its rapid evolutionary adaptation. This review consolidates evidence from the past decade on AIV-autophagy interactions, emphasizing mechanistic insights and therapeutic potential. Research indicates that various AIV strains can trigger autophagosome formation via viral components, although the completeness of autophagic flux is not fully understood. These virus–host interactions are notably influenced by viral genotypes (e.g., H5N1 vs. H9N2) and host species (avian vs. mammalian). Current studies suggest that modulating autophagy may reduce AIV-induced acute lung injury, with pharmacological agents showing potential in mitigating inflammatory responses. We systematically explore three research areas: (1) strain-specific mechanisms of autophagy induction, (2) host-specific autophagic responses in poultry and human models, and (3) the therapeutic potential of stage-specific autophagy manipulation. This synthesis clarifies critical knowledge gaps, particularly the need for standardized autophagic flux assessment in avian cells, while providing a conceptual framework for developing autophagy-targeted strategies against AIV pathogenesis.

## 1. Introduction

The avian influenza virus (AIV) is the causative agent of avian influenza, mainly affecting avian species but also capable of infecting humans and other mammals, making it a significant zoonotic pathogen with global public health implications [1]. Since the 1990s, highly pathogenic AIV strains, like H5N1 and H7N9, have caused numerous outbreaks worldwide, posing major public health risks [2,3]. Notably, H5N1 has a human mortality rate of over 30%, and H7N9 also has a relatively high mortality rate [4]. Recently, there has been an increase in the frequency and severity of AIV transmission, especially with the emergence of the H10N3 subtype, highlighting its strong ability to mutate and adapt [5].

Autophagy functions as a vital defense mechanism within host cells, playing a key role in the metabolism of intracellular materials, the removal of damaged organelles, and the enhancement of the host’s antiviral capabilities through the recognition and degradation of viral particles [6]. Studies have shown that AIVs can trigger an autophagic response in host cells, which may aid in their replication and survival by modulating the autophagy pathway [7]. Consequently, elucidating the interactions between the AIV and the autophagy pathway is essential for advancing our understanding of host antiviral defenses and exploring potential clinical applications.

Within this framework, this article investigates the interactions between AIVs and autophagy, evaluates the role of autophagy in the viral life cycle, and explores the interrelationships among the virus, autophagy, and host immune responses. The objective is to offer novel insights and concepts for future research and the development of therapeutic strategies.

## 2. Characteristics of Avian Influenza Virus

### 2.1. Genotyping and Molecular Structure

AIV belongs to the Orthomyxoviridae family and is characterized by a genome comprising eight segments of negative-strand RNA that encode a range of structural and non-structural proteins. AIVs are classified into various subtypes based on the diverse combinations of their surface glycoproteins, hemagglutinin (HA), and neuraminidase (NA), including subtypes such as H3 [8], H5 [9], H6 [10], H7 [3], H9 [11] and H10 [12]. The pathogenicity of AIV in chickens allows for its categorization into highly pathogenic avian influenza virus (HPAIV) and low pathogenic avian influenza virus (LPAIV) [13]. HPAIV predominantly encompasses the H5 and H7 subtypes, which cause severe disease in avian species, often leading to mortality rates in poultry approaching 100%, with a potential for cross-species transmission [14]. In contrast, LPAIV primarily encompasses subtypes such as H3, H9, and H10 [8,11,12]. Although the associated mortality rate is comparatively lower, LPAIV can compromise the immune system of infected poultry, thereby increasing susceptibility to secondary infections that pose significant threats to poultry production and lead to substantial economic losses. Genomically, the HA and NA genes of AIV exhibit considerable variability, which enhances the virus’s adaptability and pathogenicity across different host species. Notably, specific mutations linked to the pathogenicity and host adaptability of the virus have been identified in the genomes of the H5N1 and H7N9 subtypes [15,16].

### 2.2. Key AIV Viral Proteins and Their Targeted Autophagy Regulatory Stages

**Figure 1:**
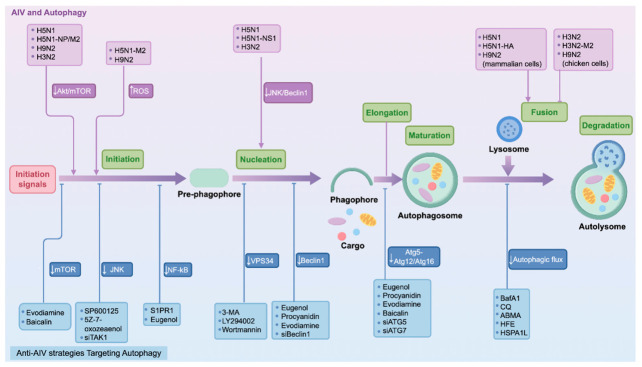
Regulation of autophagy by different subtypes of AIVs and anti-AIV strategies targeting autophagy (by Figdraw: https://www.figdraw.com/, acccessed on 25 February 2025).

The influenza virus is capable of encoding multiple functional viral proteins that interact with the host’s autophagy machinery, thereby precisely manipulating each core stage of the conserved autophagic catabolic process to promote its own replication and evade the immune response [7]. Autophagy progresses sequentially through distinct stages: initiation, nucleation, elongation and closure, fusion, and degradation, each characterized by specific core functional complexes [17], all of which are vulnerable to intervention by AIV proteins (Figure 1). HA induces endoplasmic reticulum stress via the PERK-eIF2α pathway, triggering autophagy and providing membrane materials for viral assembly [18]. The nucleoprotein (NP) and matrix protein 2 (M2) also promote autophagy by co-localizing with and interacting with LC3 [19,20]. The nucleation stage is dependent on the Beclin1-VPS34-ATG14L complex for the production of phosphatidylinositol 3-phosphate (PI3P), which is a primary target of the non-structural protein 1 (NS1) [21]; NS1 also binds to and sequesters Beclin1, thereby interfering with autophagosome formation [21]. During the elongation and closure phases of autophagosome formation, the AIV M2 protein contains an LC3-interacting region (LIR) motif that directly binds to LC3-II, anchoring viral assembly sites on autophagosomes [19,22]. During the fusion stage of mature autophagosomes and lysosomes, a process also referred to as autophagic flux, AIVs predominantly depend on M2 to regulate this fusion process [23].

## 3. Regulation of Autophagy by Different Subtypes of AIVs

AIVs are classified into various subtypes based on their surface antigens, including H5N1, H9N2, H7N9, and H3N2. These subtypes exhibit considerable variability in their regulatory effects on the autophagy pathways of different host cells, influenced by both the viral subtypes and the host cell types. For instance, in avian cells, the H5N1 virus typically enhances its replication capacity by inducing autophagy, whereas H9N2 does not significantly trigger autophagy [21]. Empirical studies have demonstrated that H5N1 infection stimulates the formation of autophagosomes and autolysosomes [24], whereas H3N2 infection promotes autophagosome formation but inhibits the fusion of autophagosomes with lysosomes [23]. Understanding these differential regulatory mechanisms provides essential insights for developing antiviral strategies tailored to specific subtypes.

### 3.1. H5N1

The H5N1 AIV subtype, noted for its high pathogenicity, demonstrates a broad host range encompassing various avian species, humans, and other mammals [25]. Empirical studies have demonstrated that H5N1 can trigger the formation of autophagosomes and enhance autophagic flux in a variety of host cell types (Figure 1 and Table 1). This autophagic process is marked by the conversion of the autophagy marker protein LC3-I to LC3-II and a reduction in p62 levels, as evidenced in human A549 [19,26] and 293T cells [24], as well as in chicken-derived chicken embryo fibroblast (CEF) and DF-1 cells [21]. Furthermore, in vivo studies have confirmed the presence of autophagosomes in the pulmonary tissues of patients and mice infected with H5N1. In contrast, such an increase in autophagosomes has not been observed in the lung tissues of patients infected with the H1N1 influenza virus [26]. Additional research has revealed that the H5N1 virus induces autophagy via the Akt-TSC2-mTOR signaling pathway in A549 cells [26]. In chicken-derived CEF or DF-1 cells, while H5N1 is capable of inhibiting the Akt-mTOR pathway, the precise regulatory mechanism of this signaling pathway on autophagy remains unclear due to the absence of commercially available antibodies specific to chicken cells [21]. In CEF, DF-1, and 293T cells, the H5N1 virus predominantly induces autophagy via the activation of the c-Jun N-terminal kinase (JNK) signaling pathway (JNK-Bcl2), which disrupts the interaction between Beclin1 and Bcl2 [21,24,27]. This mechanism is intricately associated with the regulation of TGF-β-activated kinase 1 (TAK1) [24].

The H5N1 virus modulates autophagy not only through upstream signaling pathways but also via specific viral proteins that directly impact the autophagic process (Figure 1 and Table 1). Previous research has demonstrated that H5N1 pseudotyped viral particles (H5N1pps) can initiate complete autophagy through the HA protein, as evidenced by the conversion of LC3-I to LC3-II and the degradation of p62 protein in A549 cells [18]. Investigations conducted by Wang et al. have revealed that NP and M2 encoded by H5N1 can induce autophagy by inhibiting the Akt-mTOR signaling pathway, with NP and M2 proteins co-localizing and interacting with LC3 [19]. Additionally, the NP protein has been shown to interact with heat shock protein 90 alpha 1 (HSP90A1) to modulate autophagy via the Akt-mTOR pathway in A549 cells [19]. The NS1 of H5N1 is also capable of inducing autophagy by activating the JNK pathway. In contrast, the NS1 protein encoded by H1N1 has the ability to inhibit the JNK pathway while activating the PI3K enzyme (PI3K-Akt-mTOR) signaling pathway [21]. Subsequent investigations have demonstrated that the observed variations are predominantly due to the presence of phenylalanine at position 103 in the NS1 protein of the H5N1 virus, which is pivotal in activating the JNK pathway [37]. Furthermore, the NS1 protein of H5N1 has the capability to modulate autophagy by inhibiting the Akt-mTOR signaling pathway [21]. In addition to the virus and its NS1 protein’s role in autophagy induction, the M2 protein also plays a significant role in autophagosome formation. Studies have demonstrated that the M2 protein of the H1N1 virus can impede the fusion of autophagosomes with lysosomes through its interaction with Beclin-1, thereby disrupting autophagic flux [23]. Additionally, the M2 protein encoded by H1N1 interacts with LC3, which is crucial for LC3 membrane translocation, filamentous budding, and the maintenance of viral stability [22]. According to He et al., a comparative analysis of M2 protein sequences between H5N1 and H1N1 suggests that the FVNI motif in the M2 protein of the H5N1 virus may exhibit higher affinity with LC3 [38]. Interestingly, autophagic flux was not inhibited in cells infected with the H5N1 virus. Wang et al. demonstrated that the M2 protein of H5N1 initiates the autophagy process by activating ATG5 and suppressing mTOR activity via the PI3K-Akt-mTOR signaling pathway [20]. The proton channel activity of the M2 protein is crucial for facilitating the influx of Ca^2+^ into the cytoplasm from the extracellular environment, which subsequently leads to an increase in reactive oxygen species (ROS) production. This elevation in ROS levels, either directly or indirectly, activates ATG5 and inhibits Akt and mTOR activity through the PI3K-Akt-mTOR pathway, thereby inducing autophagy mediated by the M2 protein. Simultaneously, the M2 protein impedes the formation of autolysosomes [20]. Shaotang Ye et al. have reported analogous findings, demonstrating that the M2 protein encoded by canine and avian H5N1 virus can upregulate host RAB33B expression. This upregulation facilitates autophagosome biogenesis while concurrently inhibiting autophagic flux [39]. Through the functional interaction of RAB33B-ATG16L1-LC3, the M2 protein is subsequently transported to the plasma membrane via autophagy-like vesicles, thereby maintaining efficient viral replication [39]. These observations suggest that autophagic flux may represent a finely tuned equilibrium, underscoring the complex role of autophagy in the replication of AIVs.

### 3.2. H9N2

The H9N2 avian influenza virus is categorized as a low-pathogenic AIV, predominantly circulating among wild birds and exhibiting high prevalence in poultry populations. Importantly, it has demonstrated the ability to cross species barriers and infect humans [40]. Moreover, the internal genes of the H9N2 virus contribute gene segments to other highly pathogenic AIVs, such as H5N1 and H7N9, thus playing a pivotal role in viral reassortment and the emergence of novel highly pathogenic strains [41]. H9N2 displays notable specificity in modulating autophagy across various host cells. Studies indicate that following H9N2 infection, different host cell types, including chicken embryonic fibroblasts and human cells, exhibit varied autophagic responses (Figure 1 and Table 1). In human cells, H9N2 has been shown to induce cellular autophagy, as observed in A549 cells [16,28,29,30], Human Pulmonary Microvascular Endothelial Cells (HPMECs) [28], and Human blood macrophages [31]. This induction is primarily characterized by the aggregation of GFP-LC3 puncta and increased levels of LC3 lipidation. In human blood macrophages, the H9N2/G1 virus induces autophagy more effectively than the H1N1 virus. This is evidenced by higher conversion efficiency from LC3-I to LC3-II, elevated expression levels of Beclin1, a more substantial reduction in p62 protein levels, and decreased phosphorylation levels of p70S6K [31]. Furthermore, the H9N2 virus is capable of inducing autophagosome formation in Madin-Darby canine kidney (MDCK) cells, which are derived from canines [33]. In murine infection models, H9N2 also promotes autophagy in lung tissue [29]. Conversely, in avian-derived CEF and DF-1 cells, H9N2 (A/chicken/Shanghai/F/98) only marginally induces autophagy, with no significant changes in LC3-II and p62 levels [21]. However, recent evidence indicates that infection of DF-1 cells with H9N2 (A/duck/Nanjing/01/1999) results in a sustained increase in the LC3-II/LC3-I ratio and a concurrent steady decrease in p62 abundance [32], which may be attributed to differences in host origins. Further investigations have identified instances of impaired autophagic flux in DF-1 cells infected with the H9N2 strain (A/chicken/Shanghai/F/98) [21]. This regulation of autophagy, specific to the host cell, offers significant insights into the pathogenic mechanisms of H9N2 and its transmission across different hosts. Analogous to the H5N1 virus, H9N2 initiates autophagy by inhibiting the Akt/TSC2/mTOR signaling pathway, a process linked to oxidative stress induced by H9N2 infection [30] (Figure 1 and Table 1). Unlike H5N1 and H1N1, H9N2 infection in CEF cells leads to moderate activation of JNK and pronounced activation of PI3K, yet does not trigger a substantial downstream autophagic response [21]. Additionally, H9N2 virus infection alters intracellular lipid metabolism, increasing the levels of lipid metabolic products, which are intricately linked to the regulation of autophagy [16].

The regulation of autophagy by viral proteins encoded by H9N2 remains insufficient. Research by Liu et al. demonstrates that the avian-adapted PB2 protein from H9N2, when integrated into a recombinant H1N1 influenza virus, promotes autophagosome formation and autophagic flux in A549 cells. Moreover, the viral ribonucleoprotein (vRNP) induced by avian PB2 in infected cells co-localizes with the autophagosome marker LC3. Notably, the suppression of the autophagic flux marker protein p62 expression inhibits viral replication, a phenomenon absent in the wild-type H1N1 virus [42]. Additionally, Luo et al. have demonstrated that H9N2 PB1 mediates the autophagic degradation of the immune-related protein MAVS. The application of inhibitors such as 3-Methyladenine (3-MA) and chloroquine (CQ) has shown that the MAVS degradation mediated by H9N2 PB1 can be reversed through the autophagy pathway, indicating that H9N2 PB1 facilitates MAVS degradation via this pathway [43].

### 3.3. H3N2

Influenza viruses of the H3 subtype are characterized by low pathogenicity and are prevalent among humans, dogs, horses, and birds [44]. The H3N2 canine influenza virus (CIV), which originated in avian sources, emerged in dogs in Asia and has since become endemic [45]. Research has demonstrated that H3N2 infection in A549 and Ana-1 cells can induce autophagy by inhibiting mTOR phosphorylation, as evidenced by an increase in autophagosomes and the conversion of LC3-I to LC3-II [34] (Figure 1 and Table 1). Ren et al. corroborated these findings, showing that H3N2 infection leads to an increase in autophagosomes in HEK293 cells [46]. Notably, a study by Gannagé et al. revealed that although H3N2 induces autophagosome formation in epithelial cell lines such as A549, mouse lung epithelial cells (MLE-12), human breast carcinoma cells (MDAMC), and human keratinocyte cells (HaCat), it impedes the fusion of autophagosomes with lysosomes [23]. The inhibition observed is primarily ascribed to the M2 protein encoded by the H3N2 virus, which impedes the autophagic degradation process, leading to the intracellular accumulation of autophagosomes. Notably, the proton channel activity of the M2 protein does not play a role in this mechanism. Recent studies suggest that, analogous to the H5N1 virus, the M2 protein of H3N2 facilitates its trafficking to the plasma membrane via autophagy-like vesicles by inhibiting autophagic flux, thereby enhancing viral replication [39]. Additionally, research has demonstrated that the H3N2 subtype simultaneously activates mTORC1 and autophagy-related transcription. However, this activation circumvents the ULK1 signaling pathway, which is typical of classical autophagy, and instead promotes autophagy via the activation of the JNK signaling pathway [35], similar to mechanisms observed in H5N1.

### 3.4. Other AIVs

The H7N9 avian influenza virus is a prominent member of the highly pathogenic AIVs and has been recognized as a significant threat to human health since its initial identification in humans in 2013 [47]. This virus exhibits the capability to transmit among poultry and infect humans, highlighting its extensive host range and potential for cross-species transmission. However, research on the interaction between H7N9 and remains limited. Yang et al. conducted a transcriptomic analysis that revealed H7N9 infection can upregulate autophagy-related genes, a finding observed in human A549 cells, providing preliminary evidence that H7N9 can induce autophagy [15]. Furthermore, Sun et al. identified autophagosomes in human patients infected with H7N9 using electron microscopy [48]. In addition, the H6N6 avian influenza virus, which typically does not manifest obvious clinical symptoms, possesses a broader host range [49]. Research by Yang et al. indicates that H6N6 infection can also induce autophagy in RAW264.7 cells, primarily evidenced by the lipidation of LC3 and the increased presence of autophagosomes as observed through electron microscopy [36] (Figure 1 and Table 1).

### 3.5. Comparative Analysis Across AIV Subtypes

All AIV subtypes are capable of commandeering host autophagic pathways, employing conserved molecular mechanisms to facilitate viral replication. Predominant strains, such as H5N1, H9N2, and H3N2, are adept at inducing autophagosome formation and promoting the lipidation transition from LC3-I to LC3-II within host cells [19,21,28,34]. Regarding signaling pathways, most subtypes initiate autophagy through the inhibition of the Akt-mTOR pathway [26,30,34]. Additionally, both H5N1 and H3N2 are able to activate the JNK signaling pathway, thereby modulating cellular autophagic responses [21,35]. The M2 protein, a pivotal regulatory viral protein, impedes autophagic flux during H5N1 and H3N2 infections by being transported to the plasma membrane via autophagy-like vesicles, thus maintaining effective viral replication [20,23,39]. Various structural and non-structural proteins, including HA, NP, NS1, and PB, are involved in the modulation of autophagy, with certain viral proteins directly interacting with LC3 to influence autophagic progression [18,19,21,42,43]. Overall, the regulation of autophagy mediated by all AIV subtypes demonstrates notable specificity for host cells.

AIV subtypes exhibit distinct regulatory mechanisms, characterized by significant variations in autophagic flux, activated signaling pathways, and the functional roles of viral proteins. In terms of autophagic flux modulation, the H5N1 subtype facilitates complete autophagic flux and normal autolysosome formation across various cell types [18,21,26]. Conversely, the H3N2 subtype impedes the fusion of autophagosomes with lysosomes, resulting in substantial intracellular accumulation of autophagosomes [23]. The H9N2 subtype demonstrates notable host-specific regulation of autophagy, with divergent autophagic flux patterns observed among different viral strains. Specifically, chicken-origin H9N2 strains inhibit autophagic flux in DF-1 cells [21], whereas duck-origin strains enhance autophagic flux within the same cell line [32]. Variations are also evident in the activation patterns of signaling pathways. The H5N1 subtype primarily induces autophagy via the JNK-Bcl2 and TAK1-associated pathways [21,24]. In avian cells, the H9N2 subtype weakly activates the JNK pathway but robustly stimulates the PI3K pathway, with its autophagic modulation closely linked to intracellular lipid metabolism [21]. The H3N2 subtype triggers ULK1-independent non-canonical autophagy [35]. Furthermore, viral proteins exhibit subtype-specific biological functions. The NS1 protein of the H5N1 subtype activates the JNK signaling pathway, demonstrating effects contrary to those observed with the NS1 protein of the H1N1 subtype [21]. In the H9N2 subtype, the PB1 protein mediates the autophagic degradation of the immune adaptor MAVS, whereas the PB2 protein promotes the progression of autophagic flux [43]. Notably, the inhibitory effect of the M2 protein from the H3N2 subtype on autophagic degradation operates independently of its proton channel activity [42].

## 4. Cross-Regulation of Autophagy and Host Immunity in AIV-Infected Cells

Following the pre-treatment of macrophages derived from human blood with 3-MA or their transfection with siATG5, there was a notable reduction in the mRNA and protein levels of CXCL10 and IFN-α induced by AIV infection. Notably, while 3-MA acts as an autophagy inhibitor and influences the expression of CXCL10 and IFN-α, it does not affect the replication of H9N2/G1 [31]. Further investigations have demonstrated that the inhibition of autophagy in human cells and mouse lung tissue can significantly diminish the expression of inflammatory factors and chemokines induced by H9N2 virus through the Akt-mTOR, NF-κB, and MAPK signaling pathways. This suggests that autophagy plays a crucial role in the inflammatory response [29]. A similar observation was made in cells infected with H5N1. Research conducted by Pan et al. revealed that autophagy is associated with lung inflammation induced by H5N1pps, and there exists a positive feedback loop between H5N1pps-induced NF-κB activation and autophagy. This is primarily evidenced by the association between autophagy inhibition (via 3-MA, siBeclin1, siATG5) and reduced IL-1b, IL-6, TNF-a, CCL2, CCL5, IL-8, and CXCL2. Subsequent investigations have elucidated that autophagy modulates the inflammatory response via the NF-κB and p38-MAPK signaling pathways [18]. In A549 cells with overexpressed S1PR1, both autophagy and H5N1 virus replication were inhibited, concomitant with a decrease in the expression levels of inflammatory factors. This observation suggests that S1PR1 may modulate virus replication and the inflammatory response through the regulation of autophagy. Moreover, the activation of S1PR1 was found to inhibit the NF-κB signaling pathway, thereby reducing phosphorylation and nuclear translocation of p65, which further suppressed autophagy and the inflammatory response [28]. These findings underscore a significant association between autophagy and interferons, as well as the upstream inflammatory factors of NF-κB. Consequently, autophagy emerges as a promising immunomodulatory target in the context of AIV infection. Notably, a specific autophagy-inducing peptide, designated as AIP-C5 and developed by Sayedahmed et al., has been shown to enhance the efficacy of influenza vaccines. This enhancement is evidenced by the upregulation of genes such as tumor necrosis factor, interferon-γ, BH3-interacting domain death agonist, and immune-related GTPase family member 1. When administered alongside influenza vaccines of various strains, AIP-C5 provides enhanced protection in mice exposed to H5N2, H7N9, and H9N2 viruses [50]. This observation underscores the critical role of autophagy in immune regulation during viral infections.

MAVS serves as a critical intermediary linking autophagy and immune responses. Research conducted by Zeng et al. demonstrates that the PB1 protein from the H7N9 influenza virus facilitates K27-linked ubiquitin modification of MAVS, recruits the selective autophagy receptor NBR1, and targets MAVS for degradation within autophagosomes [51]. This process subsequently inhibits the innate immune response mediated by MAVS within the RIG-I-MAVS signaling pathway. Similarly, the PB1 protein encoded by H9N2 exhibits analogous functionality, promoting MAVS degradation via the autophagy pathway and thereby suppressing the RIG-I signaling pathway [43]. Furthermore, H7N9 attenuates the interaction between HFE and p62 by downregulating HFE expression, thereby reducing HFE-p62-mediated autophagic degradation of MAVS. This results in the stabilization of MAVS, enhances type I interferon signaling, and inhibits H7N9 replication [52]. Additionally, the H5N1-M2 protein impedes autophagosome formation, leading to decreased degradation of MAVS aggregates and an augmented host innate immune response, characterized by increased production of type I interferons and pro-inflammatory cytokines [20].

Overall, autophagy plays a pivotal role in mediating AIV-induced inflammatory and interferon responses through the Akt-mTOR, NF-κB, and MAPK pathways, with autophagy inhibition significantly reducing the secretion of inflammatory mediators. Viral proteins from various AIV subtypes modulate host innate immunity by influencing the autophagic degradation of MAVS and altering the activity of immune signaling pathways. Autophagy-related molecules and inducible peptides have the potential to disrupt viral replication and modulate host immune responses, offering promising prospects for applications in anti-influenza immunomodulation.

## 5. Anti-AIV Strategies Targeting Autophagy

### 5.1. Antiviral Drugs Targeting Autophagy

As shown in Figure 1 and Table 1, 3-MA functions as a phosphoinositide PI3K inhibitor, which impedes autophagosome formation by targeting PI3K, thus obstructing the initial stages of autophagy [53]. A substantial body of research has demonstrated that 3-MA exhibits antiviral activity against AIV both in vitro and in vivo, and it mitigates lung damage resulting from AIV infection. The study by Sun et al. provides evidence that 3-MA effectively inhibits the replication of the H5N1 virus in A549 cells and murine models, concurrently reducing acute lung injury associated with H5N1 infection [26]. Similar outcomes were observed in the treatment of lung inflammation induced by H5N1pps in mice [18]. Further investigations have revealed that 3-MA, in conjunction with LY294002 (another PI3K inhibitor), disrupts the early stages of the viral life cycle of AIV or its RNA synthesis by inhibiting autophagy [2]. Additionally, 3-MA has been shown to inhibit the replication of the H9N2 virus and alleviate pathological damage to lung tissue in H9N2-infected mice, thereby decreasing pulmonary edema and reducing inflammation and thickening of the alveolar walls [29]. Subsequent studies indicate that 3-MA and LY294002 also inhibit the replication of H9N2 virus by reducing oxidative stress in cells [30]. Additionally, the early autophagy inhibitor Wortmannin has been demonstrated to suppress H9N2 virus replication by inhibiting autophagy. This suppression does not affect the initial viral entry phase but primarily influences the viral life cycle by regulating the synthesis of M1 and M2 proteins in A549 cells [33].

In contrast, inhibitors targeting late-stage autophagy, such as bafilomycin A and CQ, predominantly impede autophagic degradation (Figure 1 and Table 2). CQ has been shown to inhibit the replication of both H9N2 and H5N1 viruses and to mitigate acute lung injury caused by viral infections in murine models [29,32,54]. Similarly, bafilomycin A has been reported to inhibit H5N1 virus replication in CEF cells [21]. ABMA, an inhibitor of late endosome function, has been shown to modulate autophagic flux by accumulating autophagosomes and preventing autolysosome formation, thereby affecting H3N2 virus replication in MDCK cells [55].

In addition to inhibitors that directly target the autophagic process, certain inhibitors of upstream autophagy signaling pathways have demonstrated antiviral efficacy against AIVs (Figure 1 and Table 2). The JNK signaling pathway is recognized as a critical mediator of autophagy induced by the H5N1 virus in avian cells. The JNK inhibitor SP600125 effectively impedes H5N1 virus-induced autophagy and replication in DF-1 cells [21]. In contrast, in A549 and NHBE cells infected with the H3N2 virus, non-classical autophagy is induced via the JNK pathway. The application of a JNK inhibitor markedly reduces H3N2 protein synthesis and autophagy pathway activation [35]. Notably, NHBE cells exhibit greater sensitivity to JNK inhibition compared to A549 cells. Antiviral strategies targeting non-classical autophagy pathways may offer reduced toxicity in vivo. Furthermore, the specific inhibitor 5Z-7-oxozeaenol, which targets the JNK pathway-associated factor TAK1, significantly obstructs H5N1 virus-induced LC3 lipidation and p62 degradation, thereby diminishing H5N1 replication in 293T cells [24].

Beyond the established autophagy inhibitors, certain natural compounds have also demonstrated efficacy in inhibiting AIV replication through modulation of the autophagy process (Figure 1 and Table 2). Baicalin, a flavonoid compound derived from the dried roots of *Scutellaria baicalensis* Georgi of the *Lamiaceae* family, has been shown in studies to reduce viral titers by inhibiting autophagy in A549 and Ana-1 cells infected with the H3N2 virus [34]. Comparable inhibitory effects were observed in RAW264.7 cells infected with the H6N6 virus [36]. Additionally, proanthocyanidin, a subclass of flavonoids, has exhibited the capacity to inhibit AIVs replication in MDCK cells, including strains H9N2, H5N1, and H3N2, by obstructing the formation of the Atg5-Atg12/Atg16 heterotrimer and enhancing the stability of the Beclin1/Bcl2 heterodimer [56]. Eugenol, the primary active compound of *Syzygium aromaticum* L., has been found to stabilize the Beclin1/Bcl2 heterodimer, inhibit autophagy, and consequently suppress the replication of IAV strains H3N2, H9N2, and H5N1 in A549 cells [57]. Further investigations reveal that eugenol can also diminish the expression of autophagy-related genes by mitigating oxidative stress and activating the ERK, p38MAPK, and IKK/NF-kB signaling pathways [57]. Moreover, evodiamine, the major active component of *Evodia rutaecarpa* Benth., has been reported to inhibit autophagy by activating mTOR activity and preventing the formation of the Atg5-Atg12/Atg16 trimer, thereby impeding the replication of IAV strains H3N2, H9N2, and H5N1 in A549 cells [58]. Additionally, Aloe vera and its constituents have the potential to inhibit IAV-induced autophagy and suppress H3N2 replication by directly interacting with a critical site on the IAV-M2 protein [59].

### 5.2. Host Anti-Viral Factors Targeting Autophagy

In addition to pharmacological interventions, the silencing of host genes can significantly reduce autophagy levels within cells and provide a more precise targeting effect (Figure 1 and Table 3). The primary host genes involved in autophagosome formation include ATG5, Beclin1, ATG7, and LC3. Notably, siATG5 has been shown to inhibit H5N1 replication both in vivo and in vitro, as well as to mitigate acute lung injury in mice induced by infections with H5N1 or H5N1pps [18,26,29,30]. Other autophagy-related genes, such as Beclin1, ATG7, and LC3, exhibit similar inhibitory effects on AIV replication [2,18,32,33]. These genes suppress viral replication in a manner analogous to autophagy inhibitors, primarily by inhibiting the synthesis of viral proteins and RNA during the early stages of the viral life cycle [2,33]. Additionally, siATG7 also inhibits the Dicer-dependent antiviral RNAi response to suppress H9N2 replication [32].

In addition to the key genes directly implicated in the autophagic process, newly identified host genes have been found to modulate AIV replication through the regulation of autophagy (Figure 1 and Table 3). For instance, Wang et al. demonstrated that the overexpression of S1PR1 inhibits H9N2 replication in A549 and HPMECs by suppressing autophagy [28]. The HFE protein facilitates the degradation of MAVS via its interaction with p62, thereby inhibiting type I interferon expression and promoting H7N9 replication [52]. Another host protein, HSPA1L, directly interacts with the H5N1 NA protein and induces NBR1-mediated autophagic degradation of NA, thereby restricting viral replication. Overexpression of HSPA1L also suppresses the proliferation of H3N2, H9N2 and H7N9 in HEK293T cells [60]. Furthermore, research conducted by Pissawong et al. resulted in the development of a human single-chain variable fragment (HuScFv) that specifically binds to the M2 protein of the H5N1 virus. Molecular docking and homology modeling suggest that this protein interacts with the anti-autophagy region of M2, potentially influencing H5N1 replication by modulating autophagy [61]. Like TAK1 inhibitors, siTAK1 significantly impedes LC3 lipidation and p62 degradation induced by the H5N1 virus [24].

Overall, targeting autophagy represents a promising therapeutic strategy for addressing AIV infections. This approach aims to achieve dual objectives: alleviating acute lung injury caused by excessive immune cell recruitment and cytokine storms, and reducing viral replication titers. A variety of chemical inhibitors have demonstrated efficacy against AIV. These include early autophagy inhibitors such as 3-MA and LY294002, late-stage autophagic flux blockers like CQ and bafilomycin A, and inhibitors of upstream signaling pathways, including the JNK inhibitor SP600125 and the TAK1 inhibitor 5Z-7-oxozeaenol. These compounds have been shown to suppress the replication of H5N1, H9N2, H3N2, and other subtypes, while also mitigating lung pathological damage (Table 2). Additionally, natural active compounds such as baicalin, proanthocyanidin, eugenol, and evodiamine exhibit anti-AIV effects by modulating autophagy (Table 2). They achieve this through mechanisms such as inhibiting autophagy-related gene expression, stabilizing the Beclin1/Bcl2 heterodimer, and preventing the formation of the Atg5-Atg12/Atg16 trimer. Furthermore, genetic interventions, including the silencing of critical autophagy genes (ATG5, Beclin1, ATG7) or the overexpression of host restriction factors (S1PR1, HFE, HSPA1L), precisely regulate cellular autophagy (Table 3). These manipulations disrupt viral protein and RNA synthesis and modulate host antiviral immune responses, thereby inhibiting the replication of AIV.

## 6. Conclusions and Future Perspectives

Drawing from the preceding discussion, it is evident that the interaction between AIV and autophagy is modulated by the virus’s genotype, virulence, and the host species. These interactions demonstrate variability across different viruses and cell types; however, most experimental studies have been predominantly conducted using human cells, with a limited number of investigations focusing on avian cells or animal models. This scarcity of research on avian systems restricts our understanding of the mechanisms through which AIVs infect avian hosts. Comprehending the transmission dynamics of AIVs in livestock and poultry is essential for devising effective prevention and control strategies. Consequently, it is crucial to incorporate avian cells or animal models in future research endeavors to elucidate the interactions between various AIV genotypes and autophagy.

Current evidence indicates that viruses of distinct genotypes exert differential effects on autophagic flux. However, most research has predominantly focused on the impact of the virus on the initiation phase of autophagy, particularly the early stages of autophagosome formation. There is a significant research gap regarding the role of AIVs in modulating the transport processes between autophagosomes and autophagy-related vesicles, which are critical for viral replication and release. The transport of autophagic vesicles and the fusion of autophagosomes with lysosomes are essential processes for RNA virus replication [62]. Consequently, future studies should focus on elucidating the interaction between AIVs and the downstream pathways of autophagy.

A substantial body of research indicates that autophagy holds potential as a therapeutic target for alleviating the cytokine storm associated with highly pathogenic AIV infections. Consequently, it is imperative to explore immune regulatory mechanisms that influence autophagy and to develop pharmacological interventions in this context, establishing a critical direction for future research. Moreover, the existing literature reveals a notable deficiency, with only a single study addressing the role of autophagy in influenza vaccine development, underscoring a significant gap in understanding that necessitates further investigation.

In light of advancements in biotechnologies, including multi-omics and bio-related artificial intelligence, it is crucial to investigate the development of broad-spectrum antiviral drugs that target autophagy through the utilization of omics and molecular simulation techniques. While certain novel technologies have been implemented in H1N1 research. For example, Martin-Sancho et al. employed global genetic loss-of-function screens integrated with transcriptomics and proteomics data to elucidate the cellular restriction landscape of influenza A virus [63]. Figueras-Novoa et al. resolved the three-dimensional structure of the complex composed of the C-terminal LIR of H1N1-M2 protein and autophagy-related LC3B via X-ray crystallography [64]. Nevertheless, the distinct differences between H1N1 and AIVs necessitate further investigation into the interactions between AIVs and their avian hosts.

In conclusion, autophagy serves as a central regulatory nexus that connects the biological characteristics of avian influenza viruses with host defense mechanisms, significantly impacting viral replication, immune modulation, disease pathogenesis, and the development of targeted therapies. Considering the continuous evolutionary changes in avian influenza viruses and their ongoing threats to public health, exemplified by the persistent H5N1 clade 2.3.4.4b epizootics and sporadic cross-species transmissions of emerging viral subtypes [65], a comprehensive understanding of the interaction mechanisms between viruses and autophagy is crucial for the development of innovative antiviral agents and pandemic prevention strategies. Future interdisciplinary research encompassing virology, cell biology, immunology, and structural biology is urgently needed to advance fundamental mechanistic studies, validate in vivo biological functions, and expedite the clinical translation of relevant findings.

## Figures and Tables

**Table 1:** AIV and autophagy.

Virus and Viral Protein	Effect on Autophagy	Cell and Animal	Experiment Model	Reference
H5N1pps	Induce the formation of an autophagosome and an autolysosome	A549, Mice	In vitro and in vivo	[18]
H5N1-HA				
H5N1	Induce the formation of an autophagosome	A549	In vitro	[19]
H5N1-NP				
H5N1-M2				
H5N1	Induce the formation of an autophagosome and an autolysosome	293T	In vitro	[24]
H5N1	Induce the formation of an autophagosome and an autolysosome	CEF, DF-1	In vitro	[21]
H5N1	Induce the formation of an autophagosome and an autolysosome	A549 and Mice	In vitro and in vivo	[26]
H9N2	Induce the formation of an autophagosome	A549, HPMECs	In vitro	[28]
H9N2	Induce the formation of an autophagosome	A549, Mice	In vitro and in vivo	[29]
H9N2	Induce the formation of an autophagosome	A549	In vitro	[30]
H9N2	Induce the formation of an autophagosome and an autolysosome	Human blood macrophages	In vitro	[31]
H9N2 (A/chicken/Shanghai/F/98)	Induce the formation of an autophagosome and an autolysosome	CEF, DF-1	In vitro	[21]
H9N2 (A/duck/Nanjing/01/1999)	Induce the formation of an autophagosome but inhibit autophagy flux	DF-1	In vitro	[32]
H9N2	Induce the formation of an autophagosome	MDCK	In vitro	[33]
H3N2	Induce the formation of an autophagosome	A549, Ana-1	In vitro	[34]
H3N2	Induce the formation of an autophagosome	A549	In vitro	[35]
H3N2	Induce the formation of an autophagosome but inhibit autophagy flux	A549, MLE-12, MDAMC and HaCat	In vitro	[23]
H6N6	Induce the formation of an autophagosome	RAW264.7	In vitro	[36]

**Table 2:** Antiviral drugs targeting autophagy.

Inhibitor	Mechanisms of Action	Target Virus	Cells and Animals	Experiment Model	Reference
3-MA	Inhibit autophagy via class III PI3K	H5N1pps	A549 and mice	In vitro and in vivo	[18]
		H5N1	A549	In vitro and in vivo	[26]
		H5N1	A549	In vitro	[19]
		H9N2	A549 and mice	In vitro and in vivo	[29]
		H9N2	A549	In vitro	[30]
		H9N2	Human blood macrophages	In vitro	[31]
		H9N2	MDCK	In vitro	[33]
LY294002	Inhibit autophagy via PI3K	H5N1	A549	In vitro	[19]
		H9N2	A549	In vitro	[30]
Wortmannin	Inhibit autophagy via PI3K	H9N2	MDCK	In vitro	[33]
CQ	Inhibit autophagic flux	H9N2	A549 and mice	In vitro and in vivo	[29]
		H5N1, H9N2	CEF and DF-1	In vitro	[21]
		H9N2	DF-1	In vitro	[32]
		H5N1	Mice	In vivo	[54]
bafilomycin A	Inhibit autophagic flux	H9N2	A549 and mice	In vitro and in vivo	[29]
ABMA	Inhibit autophagic flux	H3N2	MDCK	In vitro	[55]
SP600125	Inhibit autophagy through the inhibition of the JNK1 pathway	H5N1, H9N2	CEF and DF-1	In vitro	[21]
		H3N2	A549 and noncancer lung bronchial epithelial cells	In vitro	[35]
5Z-7-oxozeaenol	Inhibit autophagy through the inhibition of the TAK1-associated JNK1 pathway	H5N1	293T	In vitro	[24]
Baicalin	Inhibit autophagy-related gene expression	H6N6	RAW 264.7	In vitro	[36]
	Inhibit autophagy by increasing the phosphorylation of mTOR	H3N2	A549 and Ana-1	In vitro	[34]
Procyanidin	Inhibit autophagy by obstructing the formation of the Atg5-Atg12/Atg16 heterotrimer and enhancing the stability of the beclin1/bcl2 heterodimer	H9N2, H5N1, H3N2	MDCK	In vitro	[56]
Eugenol	Inhibit autophagy by obstructing the formation of the Atg5-Atg12/Atg16 heterotrimer and enhancing the stability of the beclin1/bcl2 heterodimer	H3N2, H9N2, H5N1	A549	In vitro	[57]
Evodiamine	Inhibit autophagy by obstructing the formation of the Atg5-Atg12/Atg16 heterotrimer and enhancing the stability of the beclin1/bcl2 heterodimer	H3N2, H9N2, H5N1	A549 cells	In vitro	[58]

**Table 3:** Host anti-viral factors targeting autophagy.

Inhibitor	Mechanisms of Action	Target Virus	Cells and Animals	Experiment Model	Reference
siATG5	Inhibit autophagosome formation	H5N1pps	A549/Mice	In vitro and in vivo	[18]
	Inhibit autophagosome formation	H5N1	A549	In vitro and in vivo	[26]
	Inhibit autophagosome formation	H5N1	A549	In vitro	[19]
	Inhibit autophagosome formation	H9N2	A549 and mice	In vitro and in vivo	[29]
	Inhibit autophagosome formation	H9N2	A549	In vitro	[30]
	Inhibit autophagosome formation	H9N2	Human blood macrophages	In vitro	[31]
	Inhibit autophagosome formation	H9N2	MDCK	In vitro	[33]
siBeclin1	Inhibit autophagosome formation	H5N1pps	A549/Mice	In vitro and in vivo	[18]
	Inhibit autophagosome formation	H5N1	A549	In vitro	[19]
	Inhibit autophagosome formation	H9N2	MDCK	In vitro	[33]
siATG7	Inhibit autophagosome formation	H5N1	A549	In vitro	[19]
	Inhibit the interaction between ATG7 and dicer	H9N2	DF-1	In vitro	[32]
S1PR1 overexpression	Inhibit autophagy through NF-κB signaling	H9N2	A549 and HPMECs	In vitro	[28]
siTAK1	Inhibit autophagy through the inhibition of the TAK1-associated JNK1 pathway	H5N1	293T	In vitro	[24]
HFE overexpression	induce the degradation of MAVS via the interaction between HFE and p62	H7N9	BMDMs and mice	In vitro and in vivo	[52]
HSPA1L overexpression	induce NBR1-mediated autophagic degradation of NA	H5N1/H3N2/H9N2/H7N9	HEK293T cells	In vitro	[60]

## Data Availability

No new data were created or analyzed in this study. Data sharing is not applicable to this article.
